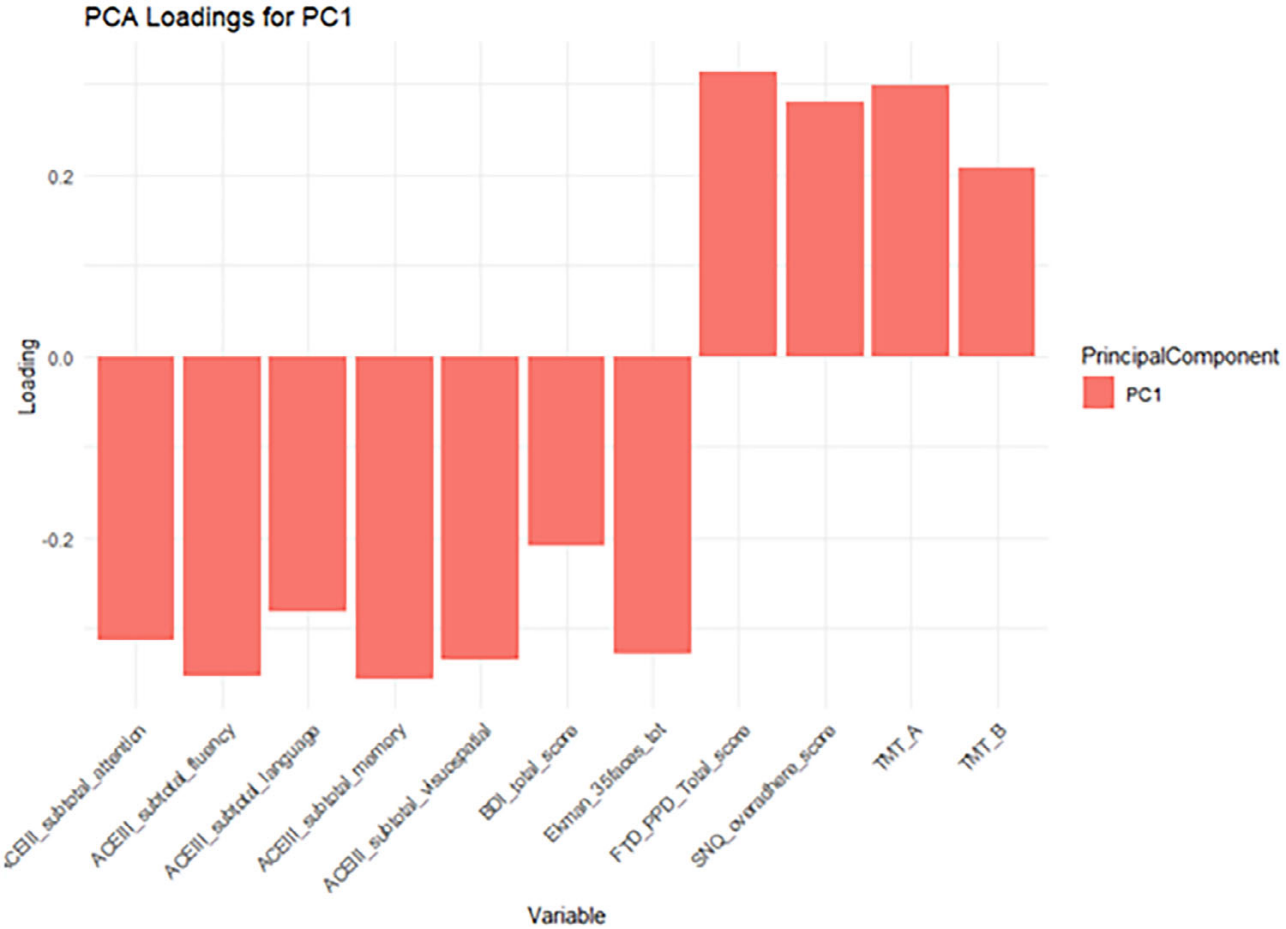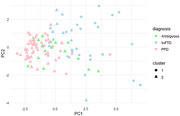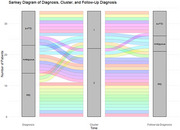# Predicting follow‐up diagnosis of individuals presenting with uncertain sporadic bvFTD diagnosis

**DOI:** 10.1002/alz70857_106357

**Published:** 2025-12-26

**Authors:** Sterre C.M. de Boer, Simon Ducharme, Chiara Fenoglio, Willem L. Hartog, Dirk N. van Paassen, Flora H. Duits, Emma Weltings, Giorgio G Fumagalli, Lina Riedl, Sophie Matis, Zac Chatterton, Ishana Rue, Ramon Landin‐Romero, Patrick Sommer, Timo Grimmer, Daniela Galimberti, Glenda M Halliday, Olivier Piguet, Yolande A.L. Pijnenburg

**Affiliations:** ^1^ Alzheimer Center Amsterdam, Department of Neurology, Amsterdam Neuroscience, Vrije Universiteit Amsterdam, Amsterdam UMC, Amsterdam, Netherlands; ^2^ Amsterdam Neuroscience, Neurodegeneration, Amsterdam, Noord‐Holland, Netherlands; ^3^ The University of Sydney, School of Psychology and Brain & Mind Centre, Sydney, NSW, Australia; ^4^ Montreal Neurological Institute, McGill University, Montreal, QC, Canada; ^5^ McConnell Brain Imaging Centre, Montreal Neurological Institute, McGill University, Montreal, QC, Canada; ^6^ University of Milan, Fondazione Cà Granda, IRCCS Ospedale Policlinico, Milan, Italy; ^7^ Department of Biomedical, Surgical and Dental Sciences. University of Milan, Milan, Milan, Italy; ^8^ Alzheimer Center Amsterdam, Neurology, Vrije Universiteit Amsterdam, Amsterdam UMC location VUmc, Amsterdam, Netherlands; ^9^ Amsterdam Neuroscience, Neurodegeneration, Amsterdam, Netherlands; ^10^ Alzheimer Center Amsterdam, Neurology, Vrije Universiteit Amsterdam, Amsterdam UMC, Amsterdam, Netherlands; ^11^ University of Trento, Rovereto, Italy, Rovereto, Italy; ^12^ Marion von Tessin‐Memory Zentrum, Munich, Germany; ^13^ The University of Sydney, Brain and Mind Centre, Sydney, NSW, Australia; ^14^ The University of Sydney, Brain & Mind Centre, Camperdown, NSW, Australia; ^15^ Douglas Mental Health University Institute, McGill University, Montreal, QC, Canada; ^16^ Technical University of Munich, School of Medicine and Health, TUM University Hospital, Center for Cognitive Disorders, Munich, Bavaria, Germany; ^17^ Klinikum rechts der Isar, Technical University of Munich, School of Medicine, Munich, Germany; ^18^ University of Milan, Milan, MI, Italy; ^19^ Fondazione IRCCS Ca’ Granda, Ospedale Maggiore Policlinico, Milan, MI, Italy; ^20^ The University of Sydney, Sydney, NSW, Australia; ^21^ Alzheimer Center Amsterdam, Department of Neurology, Amsterdam UMC, location VUmc, Amsterdam, Netherlands; ^22^ Amsterdam Neuroscience, Amsterdam UMC, Amsterdam, Netherlands

## Abstract

**Background:**

Delays in the diagnosis of sporadic behavioral variant of frontotemporal dementia (s‐bvFTD) are hindering clinical care and trial enrolment. This delay is attributed to the clinical heterogeneity of s‐bvFTD and its overlap with primary psychiatric disorders (PPD). The DIPPA‐FTD consortium aims to improve early diagnosis by including individuals with late‐onset behavioral change with ambiguous diagnoses that might turn out to be s‐bvFTD. Here, we aimed to predict the follow‐up diagnosis of these ambiguous cases by applying principal component analysis (PCA) from baseline clinical assessment.

**Method:**

In a subset (ambiguous=16, s‐bvFTD=33, PPD=57) of the ongoing DIPPA‐FTD study (de Boer et al., JAD:2024;97(2):963‐973), We applied PCA to baseline clinical data, including Addenbrooke's Cognitive Examination‐III (ACEIII), Beck Depression Inventory‐II (BDI‐II), Ekman‐35, FTDvsPPD Checklist, Social Norm Questionnaire (SNQ) and Trail Making Test A+B (TMT). We compared Principal Components (PCs) between diagnostic groups, and associations with clinician‐rated diagnostic certainty were assessed. Optimal number of k=2 for final clustering was determined using the elbow method on k‐means clustering of all 11 PCs. Data‐driven clusters and follow‐up diagnosis were used to evaluate diagnostic prediction accuracy.

**Result:**

The first principal component (PC1) explained 43.8% of the variance. Loadings of PC1 are shown in Figure 1. Significant differences were found in PC1 scores between bvFTD, PPD, and Ambiguous cases (all *p* <0.05, adjusted for multiple testing). Cluster 1 (PC1 mean sore 2.76) consisted predominantly of s‐bvFTD cases (78.1%), while cluster 2 (PC1 mean score ‐1.19) predominantly consisted of PPD cases (73.0%). PC1 and PC2 were selected for a cluster plot (Figure 2). Higher PC1 scores correlated with greater diagnostic certainty for s‐bvFTD, while lower PC1 scores indicated higher certainty for PPD (*r* = 0.74, *p* <0.001). Among *n* = 34 thus far known follow‐up cases, six ambiguous cases from cluster 2 switched to PPD after one year; two ambiguous cases in cluster 1 and one ambiguous case in cluster 2 remained ambiguous at follow‐up; none switched to s‐bvFTD (see Figure 3).

**Conclusion:**

In this pilot, a data‐driven approach identified baseline profiles for s‐bvFTD and PPD, potentially aiding in an early accurate diagnosis of individuals presenting with late‐life behavioral change.